# Quantitative description on structure–property relationships of Li-ion battery materials for high-throughput computations

**DOI:** 10.1080/14686996.2016.1277503

**Published:** 2017-02-14

**Authors:** Youwei Wang, Wenqing Zhang, Lidong Chen, Siqi Shi, Jianjun Liu

**Affiliations:** ^a^ State Key Laboratory of High Performance Ceramics and Superfine Microstructure, Shanghai Institute of Ceramics, Chinese Academy of Sciences, Shanghai, PR China; ^b^ School of Materials Science and Engineering, Shanghai University, Shanghai, PR China; ^c^ Materials Genome Institute, Shanghai University, Shanghai, PR China

**Keywords:** Li-ion battery materials, high throughput screening, quantitative description, structure-property, 206 Energy conversion / transport / storage / recovery, 404 Materials informatics / Genomics

## Abstract

Li-ion batteries are a key technology for addressing the global challenge of clean renewable energy and environment pollution. Their contemporary applications, for portable electronic devices, electric vehicles, and large-scale power grids, stimulate the development of high-performance battery materials with high energy density, high power, good safety, and long lifetime. High-throughput calculations provide a practical strategy to discover new battery materials and optimize currently known material performances. Most cathode materials screened by the previous high-throughput calculations cannot meet the requirement of practical applications because only capacity, voltage and volume change of bulk were considered. It is important to include more structure–property relationships, such as point defects, surface and interface, doping and metal-mixture and nanosize effects, in high-throughput calculations. In this review, we established quantitative description of structure–property relationships in Li-ion battery materials by the intrinsic bulk parameters, which can be applied in future high-throughput calculations to screen Li-ion battery materials. Based on these parameterized structure–property relationships, a possible high-throughput computational screening flow path is proposed to obtain high-performance battery materials.

## Graphical abstract

**Figure:**
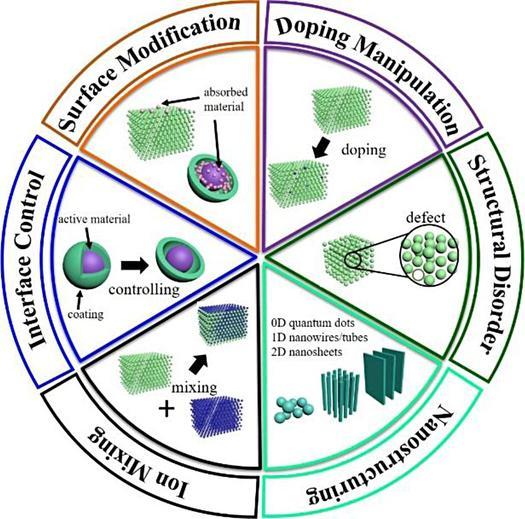

In this review, we discuss some quantitative descriptions of structure–property relationships by the intrinsic bulk parameters, which can be applied in the future high-throughput computational screening to obtain high-performance Li-ion battery materials.

## 1. Introduction

Conventional Li-ion batteries have contributed to commercial success in portable electronics, and may show increasing importance in the electric transportation market and large-scale power grids [1–5]. For such applications, new advances in performance, costs and safety are necessary, and this requires a better understanding of the known battery materials and/or the discovery of new materials to ensure a leap forward in performance. The Material Genome Initiative (MGI) provides a strategy to elucidate structure–property relationships and discover new high-performance materials by the integration of computing, experimental and data platforms [6], in which high-throughput computational and experimental screening techniques should substitute the traditional trial-and-error method in materials design by avoiding onerous and time-consuming synthesis and characterization.

Pioneered by Ceder’s research group in Massachusetts Institute of Technology (MIT), high-throughput computational screenings by setting capacity, voltage, and volume change of bulk as criteria were applied to novel Li-ion battery materials [7]. A large database of experimental and computational data are indexed to allow free online access for further calculations and experiments [8]. However, the practical use of these data faces enormous technical and scientific challenges. Some indexed battery materials cannot be synthesized, while some materials are unstable during discharge/charge cycles or have poor electrochemical performance. This is mostly attributed to electrochemical performance difference between perfect bulk and nanostructured battery materials. In fact, most practical applications in batteries are based on nanostructured configuration rather than bulk. Moreover, many electrochemical performances such as Li-ion diffusion and electronic conduction pathways in nanoscale exhibit clearly different profiles from those in microscale. In addition, the disordered structures induced by defects or doping may further generate different electrochemical performances from those in well-ordered structures. As a result, electrochemical performances of battery materials are influenced by many factors, for example, particle size, exposed surface, interface, and disordered structures. Therefore, a practical high-throughput computational screening for Li-ion battery materials should not only consider intrinsic bulk properties but also contain comprehensive criteria such as disordered structures, particle size, surface and interface, which describe nanostructure characters.

**Figure 1.**
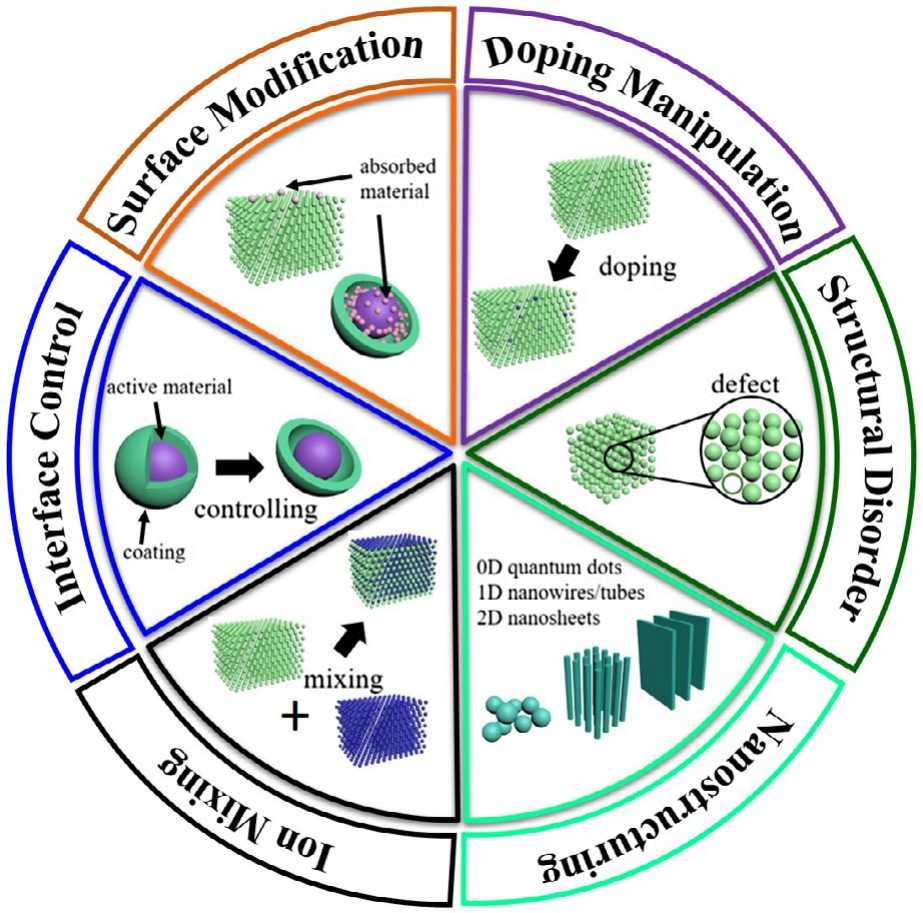
Illustration of some of the important structure–properties relationships discussed in this review.

A significant challenge is that the present high-throughput calculations for battery materials must be performed by bulk models due to expensive computational costs. Therefore, it is important to establish an effective connection between intrinsic properties of bulk materials and electrochemical performance of corresponding nanostructures. In the last 10 years, a great deal of experimental and computational studies have been performed to elucidate the various structure–property relationships involving microscopic structure, particle size, morphology, doping, phase transitions, and surface/interface effects, as shown in Figure 1. However, quantitative description of these structure–property relationships in high-throughput calculations are not established yet.

In this review, we establish quantitative descriptions for the structure–property relationships in order to perform high-throughput screenings to discover new materials and optimize known material performances. There have been some excellent reviews on this topic [3,9–11], most of which mainly discuss qualitative structure–property relationships based on underlying physical mechanisms. These summaries provide a fundamental basis for our present review.

Because of the complexity of electrochemical reactions, an effective integration of computation and experiment should be fully emphasized in the present review. Based on the established experimental information, more realistic models can be constructed to accurately describe structural stability, band structures, electron hopping, and mass transportation in Li-ion battery materials, to identify determinant factors of physical and chemical properties. Computational insights into electrochemical properties for battery materials have been discussed in previous review articles [9,10], and are beyond the scope of this review. Therefore, computations and experiments are complementary to explore the structure–property relationship of battery materials in bulk and nanostructure in order to perform high-throughput calculations.

## 2. Structure disorders

The various point defects and structural disorders under a well-ordered framework have been observed in many lithium-ion battery materials such as LiMPO_4_, Li_2_FeP_2_O_7_, LiMSiO_4_, LiMBO_3_, and LiNi_1/3_Mn_1/3_Ni_1/3_O_2_ systems [12–19]. These zero-dimension defects in cathodes are classified into antisite, Frenkel, and Schottky disorders, which are presented in Kröger–Vink notation as follows:

(1) $$\text{Antisite}:\;{\text{Li}}_{{\text{Li}}^{\times }}+{\text{M}}_{{\text{M}}^{\times }}\to {\text{Li}}_{\text{M}}^{\prime }+{\text{M}}_{\text{Li}}^{\cdot }$$

(2) $$\text{Frenkel}:\;{\text{Li}}_{\text{Li}}^{\times }\to {\text{Li}}_{i}^{\cdot }+{\text{V}}_{\text{Li}}^{\prime }$$

(3) $$\text{Schottky}:\;2{\text{Li}}_{\text{Li}}^{\times }+{\text{O}}_{\text{O}}^{\times }\to 2{\text{Li}}_{\text{M}}^{\prime }+V_{O}^{..}+{\text{Li}}_{2}\text{O}$$

where subscripts of atomic symbols (Li, O, and M = metal) and *i* represent regular lattice and interstitial positions, while superscripts (˙, ' and ˟) correspond to positive, negative and no charges, respectively [14]. In recent years, the formation energies of these point defects in the various materials were calculated based on the density functional theory (DFT), indicating that the most favorable intrinsic defect is the cation antisite defect (M↔Li). For example, charging LiMPO_4_ materials (M = Co, Fe, Mn, Ni) may generate point defects in which antisite defect is dominated [14,15,17,20]. Some low formation energies of antisite defects in Li_2_MnSiO_4_ (0.66 eV) [21,22], LiMnBO_3_ (0.75 eV) [12], LiNi_1/3_Mn_1/3_Ni_1/3_O_2_ (0.84 eV) [23], LiFePO_4_ (1.13 eV) [24], and LiMnPO_4_ (1.48 eV)[25] were calculated in the previous publications. Based on these formation energies, the concentrations of antisite defects formed by M(Fe^2+^, Mn^2+^,and Ni^2+^) and Li^+^ ions were further estimated as < 5% [12,26], which is generally consistent with experimental results, for example, 1–8% concentration of antisite defects measured by scanning transmission electron microscopy (STEM) [16,27–29].

**Figure 2.**
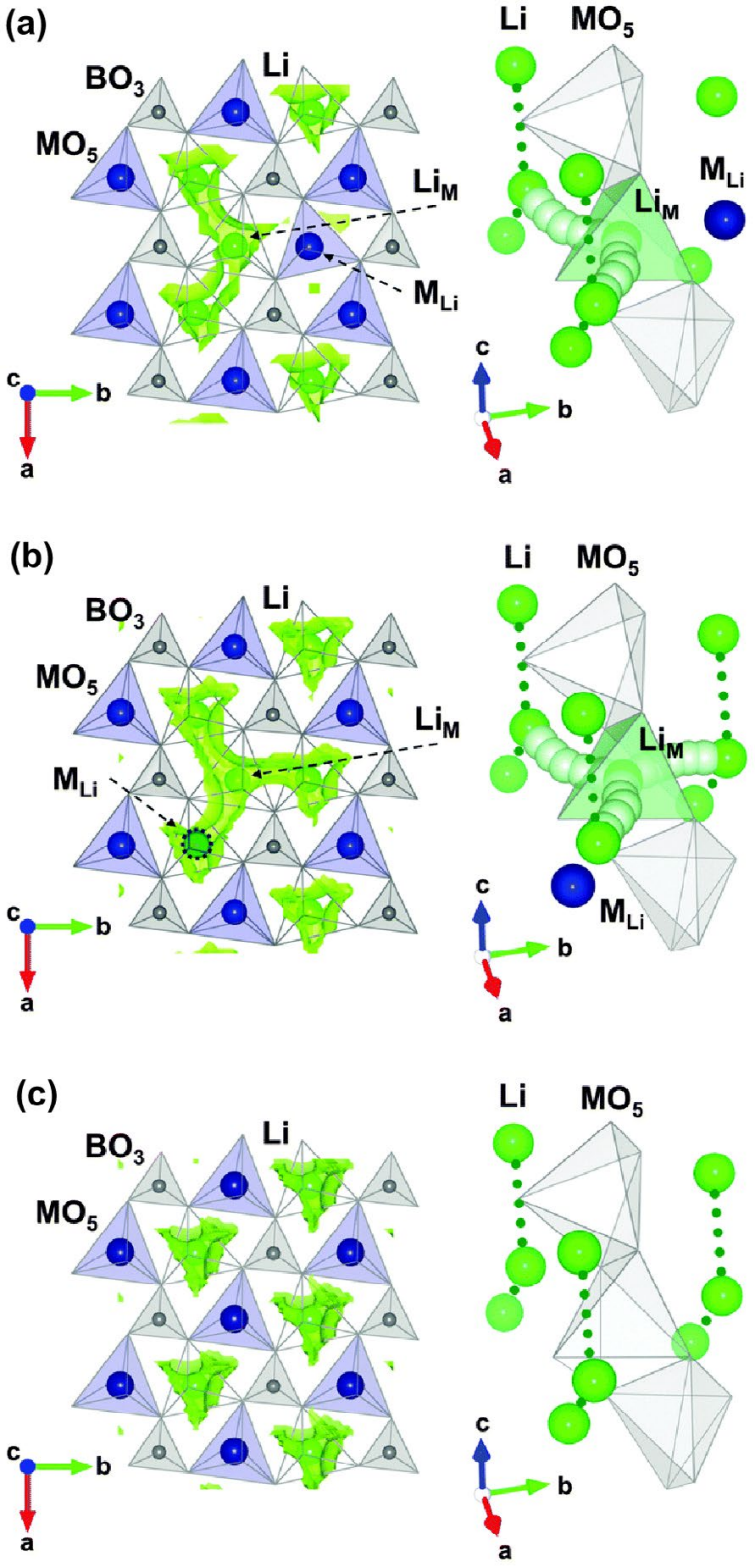
Li^+^ transport trajectory in various antisite structures in LiMn_0.5_Fe_0.375_Mg_0.125_BO_3_ based on NEB calculations. (a) The second lowest energy (Li-trapping); (b) the lowest energy antisite (Li-redirecting) and (c) antisite free structure. Reprinted from [13] with permission from the Royal Society of Chemistry.

A direct influence of antisite defect on electrochemical performance is realized by changing Li-ion migration kinetics in discharge and charge processes. On one hand, the antisite defects may generate a higher migration barrier of Li-ion and result in one-dimension migration channel blocking. Based on atomistic modeling method, Islam et al. [14,25] calculated Li-ion migration barriers of pure and antisited LiMPO_4_ (M = Mn, Fe, and Mn/Fe). Their results showed that the high antisite migration energy results in blocking effect on lithium insertion/extraction rates. On the other hand, an antisite defect can convert a 1D migration channel into a 3D-network migration path, which may reduce the 1D channel blocking effect on Li-ion migration. Kim et al. [13] performed DFT-based studies on the effect of antisite defects on the Li-ion migration in LiMn_0.5_Fe_0.4_Mg_0.1_BO_3_. Figure 2 presents local nudged elastic band (NEB) trajectory calculations for Li-diffusion in two Li_M_-M_Li_ (M = Mn, Fe, Mg) antisite and antisite-free configurations in LiMn_0.5_Fe_0.375_Mg_0.125_BO_3_. In contrast to blocking Li-ion migration channel, the antisited Li-ion provides a bridging site for crossover from one channel to others, forming 2D or 3D migration channels. In addition, two research groups separately studied defect chemistry and Li-ion migration in high-voltage cathode materials Li_2_MP_2_O_7_ (M = Co, Mn, Fe) [19, 30]. Clark et al. [30] calculated the formation energy of the Li-Fe antisite defect as 0.22 eV by atomistic modeling methods. Lee and Park [19] found such an antisite defect could lead to Li-ion migration mode change from 2D to 3D network. These studies indicate that a disordered defect with low formation energy corresponding to high concentration can extend Li-ion migration pathways.

As shown in Figure 2, the improved electrochemical performance can be attributed to a low defect formation energy in the scale of 0.4–0.5 eV and resulting in its high concentration of 11.6% in experiment (12.5% in calculation), which turns the channel-to-channel crossover to the dominant diffusion mechanism [13]. Under the assumption of low concentration of antisite defects, the concentration which depends on the defect formation energy (ΔH) can be expressed as follows:

(4) $$\text{c}\approx N_{site}\;N_{\text{config}}\;\text{exp}\left(-\frac{\Delta H}{2k_{B}T}\right)$$

where *k*_*B*_ and *T* are the Boltzmann constant and temperature, respectively, and *N*_*site*_ and *N*_*config*_ are the numbers of equilibrium sites and defect configurations, respectively. Therefore, researchers can calculate the formation energies of antisite defects and the concentrations to determine Li-ion migration mechanism. Based on Equation (4) and the previous studies, it is expected that an antisite defect with a formation energy of 0.2–0.5 eV and a concentration of ~10% can extend Li-ion migration pathways and prevent blocking of a low-dimensional pathway.

The above analysis indicates that a high concentration of antisite defects may generate a more favorable Li-ion migration mechanism by extending migration pathways and reducing migration barriers. In fact, the local structure and charge state differences of exchanged atoms play an important role in determining a formation energy of antisite defect. The local polyhedral environments of Li and transition metal have similar number and size, favoring a high concentration of antisite defects. In addition, the antisite atoms should have a similar charge state, to prevent local polarization.

For perspective, we expect that defect formation energy can be used as high-throughput calculation parameter (<0.5 eV formation energy) to screen high-performance cathode materials. One can perform fast formation energy calculations for antisite defects based on similar local occupancies, sizes, and charge states of exchanged atoms. Further defect concentration and Li-ion migration mechanism can be investigated based on the accurate first-principles calculations and experimental characterization such XRD and TEM.

## 3. Surface engineering

It is proved that tuning surface structures and morphology sometimes can allow electrochemical reactions and increase Li-ion storage capacity, which may be prevented from taking place in microscale [31–33]. In recent years surface-dependent storage capacity was reported in many publications. Li_4_Ti_5_O_12_ with spinel structure, which is of great interest as an anode material, has been applied on a large scale. What makes it attractive as a Li-ion insertion electrode is the zero-strain property, resulting in excellent cycle life: upon lithiation from initial state Li_4_Ti_5_O_12_ to its fully lithiation state Li_7_Ti_5_O_12_, there is almost no lattice change [34–36]. Also of interest is the observation of stable lithium compositions exceeding Li_7_Ti_5_O_12_ up to Li_8.5_Ti_5_O_12_ by decreasing particle size, which has been determined by neutron diffraction measurements and DFT-based calculations [37–39]. The nano-curved storage capacity is attributed to Li-ion anisotropic occupancy depending on exposed surfaces as well.

**Figure 3.**
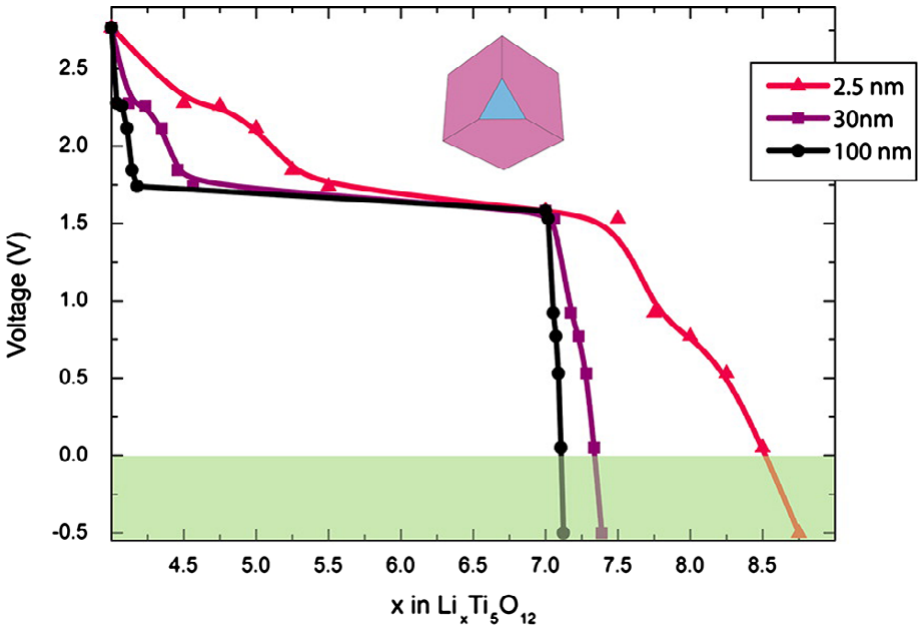
Voltage profiles for Li_7_Ti_5_O_12_ of different sizes. The red curve corresponds to a situation where Li^+^ ions enter the vacant 16c sites in Li_4_Ti_5_O_12_ and 8a sites in the (111) facet of Li_7_Ti_5_O_12_. The (111) surface is important for increasing the storage capacity. The black and purple curves correspond to particle sizes of 30 and 100 nm, respectively. Reproduced from [40] with permission of 2012 American Chemical Society.

By using first-principles methods, Ganapathy and Wagemaker [40] calculated thermodynamic properties of Li-ion inserted different surfaces of spinel Li_4+x_Ti_5_O_12_. The calculated voltages of the defective spinel Li_4_Ti_5_O_12_ reveal that it is energetically favorable to insert Li into the (100) surface leading to high voltage due to surface storage. The surface (110) with the lowest surface energy is predicted to be energetically favorable for Li-ion insertion into the vacant 16c positions. As shown in Figure 3, the (111) surface allows additional capacities that significantly exceed the bulk capacity of Li_7_Ti_5_O_12_ by occupation of 8a sites in addition to the fully occupied 16c sites. These calculated results are in agreement with experimental studies.[34–36]

In nature, the surface-dependent storage capacity increasing in Li_4+x_Ti_5_O_12_ should be attributed to the zero-strain property, i.e. very low interfacial energy and strain between the tetrahedral- and octahedral- coexisting phases. The zero-strain effect also induces a short compositional domain with constant voltage. It can be proved by no observation of two-phase separation but a solid solution for diffraction with both Li_4_Ti_5_O_12_ and Li_7_Ti_5_O_12_ phases by diffraction and nuclear magnetic resonance (NMR) measurement for 100 K micrometer-size Li_4+x_Ti_5_O_12_ [41–43]. Although surface orientations play an important role in allowing Li-ion migration between different sites (for example, 16c and 8a sites in [42]), the essence of surface-dependent storage capacity increasing still is attributed to the zero-strain effect which thermodynamically favors more accessible Li-ion occupied sites. Therefore, it is expected that different occupancy of Li-ion and strain energy of bulk materials can be used to evaluate surface-dependent storage capacity of Li-ion electrode materials.

The uniaxial strains of fully-discharged battery materials along different directions can be calculated by using supercell structure with relaxed atomic positions and cell size, by a factor of (1+*ε*), where ε is the magnitude of the strain. The strain energy difference (*E*_*diff*_) between two different directions can further been a parameter to determine surface-dependent Li^+^-occupancy and storage capacity. As a result, *E*_*diff*_ can be used as a criterion of high-throughput calculation. Besides, many Li-ion battery materials exhibit surface effects in the Li-ion migration kinetics. A typical example is LiFePO_4_ in which the two low-energy (010) and (201) surfaces were identified by DFT computations [33,44]. In addition, surface-dependent discharge voltage was also observed. DFT+U calculations exhibit that the redox potential of Li-ion insertion and extraction from (010) surface is 0.6 eV lower than from the corresponding bulk [44].

## 4. Nanosize effect

It is proved that nanosized battery materials offer several possibilities to partially improve storage capacity [40,45], discharge/charge rate [16,46–49], and lifetime of Li-ion batteries [50]. Ideally, ion diffusivity and storage capacity are intrinsic properties of the bulk materials and would not make significant change with particle sizes. However, a smaller particle generally can reduce the ionic transport path and strengthen the one-dimensional migration path, therefore improving the rate performance of a battery. A recent experimental study [51] indicated that chemical phase and fracture in Li_x_FePO_4_ depends on particle size. The lattice mismatch between LiFePO_4_ and FePO_4_ results in severe fracturing on microcrystals, whereas no mechanical damage was observed in nanosized LiFePO_4_. In this review, we focus on size-dependent Li^+^-diffusivity and phase transition.

**Figure 4.**
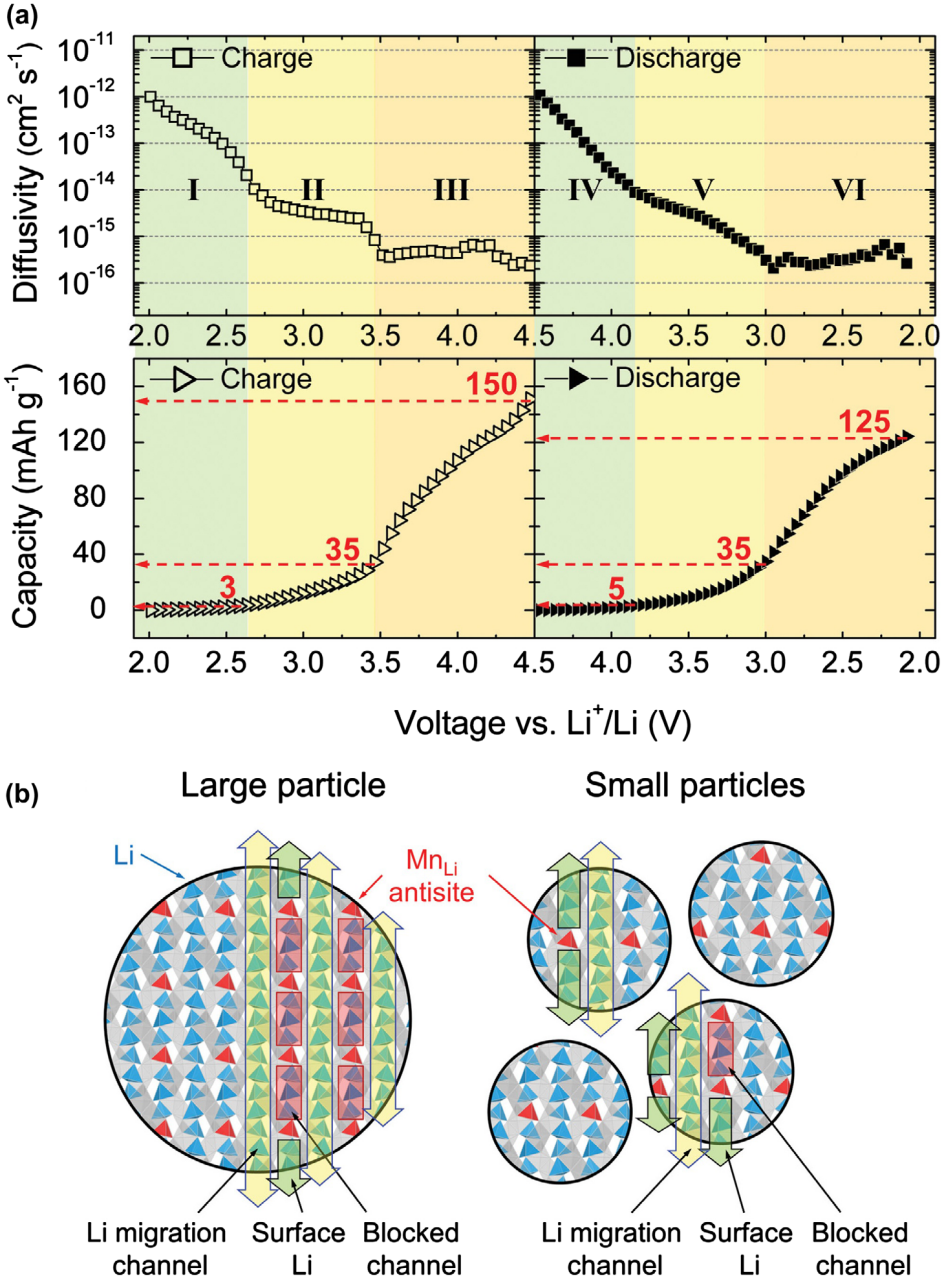
(a) Apparent Li chemical diffusivities and accumulated capacities as a function of voltage obtained by potentiostatic intermittent titration technique. (b) Schematic of size dependence of the channel blockage due to the antisite defects. Reprinted from [12] with a permission from John Wiley & Sons.

As shown in Figure 4(a), experimental and computational studies indicated that the monoclinic LiMnBO_3_ with a one-dimensional channel of Li-ion diffusion has particle-size dependent diffusivity and is less sensitive to antisite defect concentration [12]. The smaller particle size corresponds to higher Li-ion diffusion constant. Figure 4(b) presents how particle size affects the overall Li-ion mobility in the presence of the channel blocking antisite disorder. Whenever there are two or more blocking sites in a channel, the sites between them are inaccessible, removing them to enable the reversible Li capacity to increase. In fact, the same research group have reported the similar results in which the diffusion constant depends on particle size. Li-ion diffusion in bulk materials with one-dimensional atomic migration channels is much slower than that in corresponding nanoparticles [16]. Controlling particle sizes is important to improve rate performance and storage capacity of battery materials, especially for those with one-dimensional transport channel.

Although our analysis implies a particle size dependent Li^+^ kinetics, we could not establish a quantitative description on a general relationship between size and diffusion constant for high-throughput screening. On one hand, there are different migration mechanisms such as dimensionality and anisotropy in different battery materials. On the other hand, the particle size does not directly affect the diffusion constant, as shown above. Therefore, it is difficult to develop a suitable parameter as criterion for high-throughput calculations to screen high-performance battery materials.

In battery materials, structural transition, which must occur during Li-ion insertion and extraction processes, plays an important role in determining the cyclic performance of battery material. Since structural transition to undesired electrochemical phases can only occur if the particle radius *r*_*p*_ is larger than the critical nucleation radius *r*_*c*_ for that phase, it is possible to eliminate such transition by using nanoparticles with *r*_*p*_>*r*_*c*_. Therefore, small particles would easily generate the structural transition. For example, the layered LiMnO_2_ suffers from structural change during electrochemical processes and exhibits a significant capacity decrease [52].

In fact, size-dependent structural transition can be described by formation energy difference in different particle size. Further, the size-dependent formation energies of nanoparticles are approximately calculated based on the combination of bulk formation energies and surface energies of Wulff shapes, as follows:

(5) $$\Delta G_{form}\left(d,T\right)=\Delta G_{form}^{bulk}\left(T\right)\cdot V+\sum_{i}\gamma i\left(T\right)\cdot A_{i}$$

where $\Delta G_{form}^{bulk}\left(T\right)$ is the formation energy difference in bulk; *γ*_*i*_(*T*) is the surface energy of *i* facet in the calculated Wulff shape; *V* and *A*_*i*_ are the volume of particle and the *i*th surface area of Wulff shape, respectively. Therefore, the particle size *d* can be formulated by the volume and average surface area of unit volume. However, the first-principles calculations of surface energies are time consuming. Therefore, a high-throughput first-principles calculation for formation energy difference to determine particle size is not applicable in the current computer condition.

In summary, although tuning particle size plays an important role in improving electrochemical performances such as rate capacity, storage capacity, Li-ion diffusion, and lifetime, it is difficult to establish a quantitative description between size and electrochemical performance for high-throughput screening.

## 5. Doping and transition-metal mixtures

In the past decade, the doping technique has been extensively applied to improve electrical conductivity, reduce Li-ion migration barriers, and tune discharge/charge voltage of battery materials. There are a wide range of dopants used in LiFePO_4_ to substitute Li, Fe and O ions in order to improve its electrical conductivity. Since many factors such as carbon containment, doping, and phosphide formation are involved, whether electrical conductivity is enhanced by doping has stimulated a large debate [53,54]. In theory, the substitution of divalent dopants (Zn, Cu, Mg, Ca, Mn, Co) on Fe site was found to have much lower formation energies than those of monovalent (Ag), trivalent (Al, Ga) tetravalent (Ti), and pentavalent (Nb) transition metals doped on either Fe or Li sites [24,25,55]. In general, a low concentration of dopants does not significantly enhance electrical conductivity although charge compensation may occur in the aliovalent dopant.

**Figure 5.**
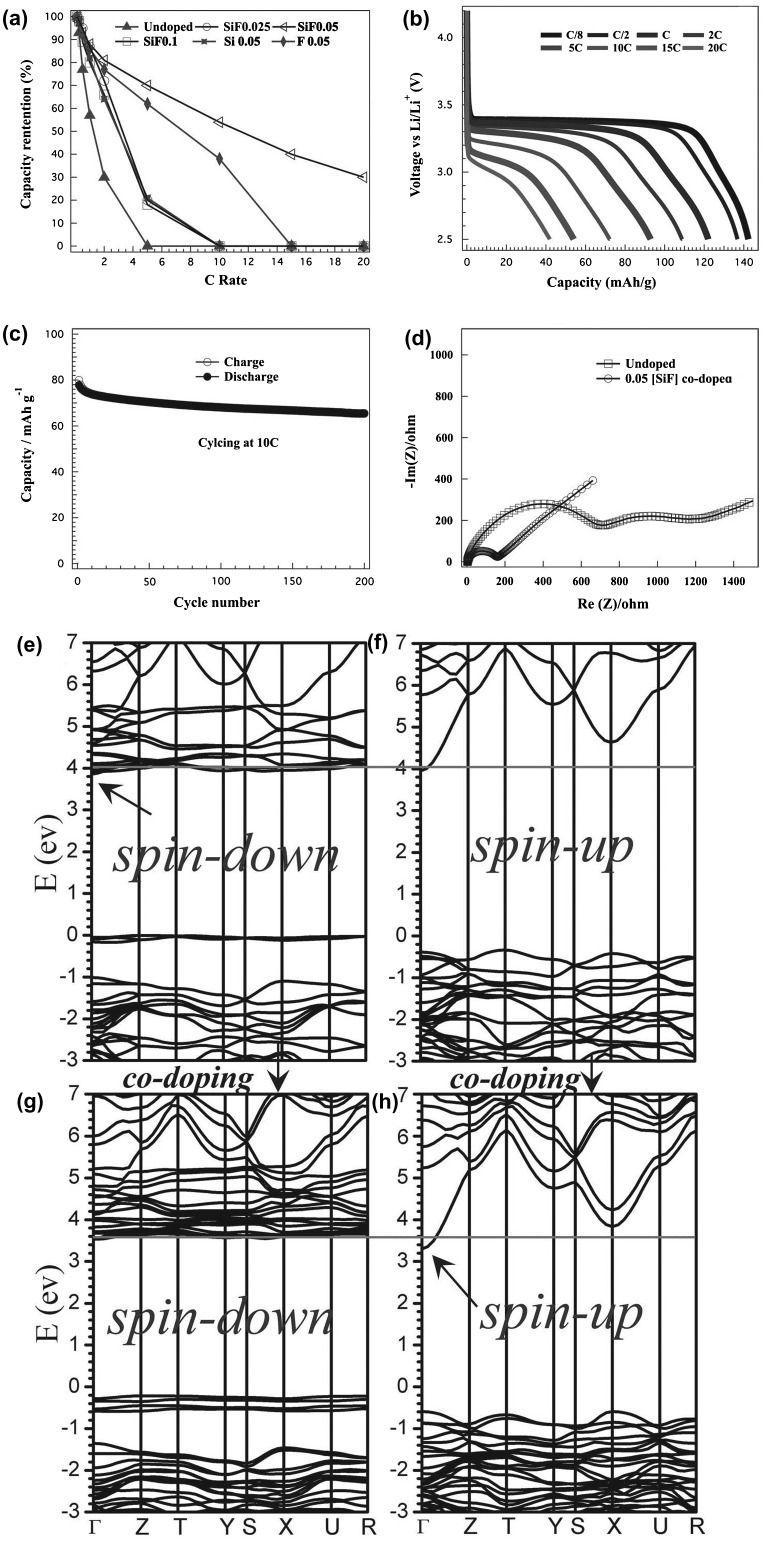
Electrochemical characterization of 0.05[Si-F] codoped LiFePO_4_. (a) Comparison of rate capabilities with other codoping levels and the single ion doping; (b) discharge voltage profiles at different rates; (c) sustainable performance at a high cycling rate of 10C; (d) comparison of electrochemical impedance spectroscopy plots for un-doped LiFePO_4_ and 0.05[Si-F] codoped LiFePO_4_. (e–h) band structures of undoped and codoped LiFePO_4_. Reprinted from [56] with permission from John Wiley & Sons.

The previous works suggested that the co-doping of F on the O sites and Si on the P sites could improve electrical conductivity of LiFePO_4_, exhibiting a 2–3 orders of magnitude increase, based on theoretical computations and materials characterizations [56–58]. As shown in Figure 5, Dillon et al. [56] explained that the doping effect modifies the nature of conduction band minimum (CBM). The transport mechanism is changed from polaron-type to band-like conduction. For the undoped LiFePO_4_, the poor electrical conductivity is attributed to very large electronic effective masses contributed by Fe-3*d* orbitals in CBM. After Si, F-codoped LiFePO_4_, the states with lighter effective mass are shifted down to form CBM, generating a higher electrical conductivity. The charge-compensating codoping approach plays an important role in improving performance of electrode materials suffering from poor electrical conductivities.

Based on the above analysis, it is expected that doping strategy can lead to higher electrical conductivity by tuning band structure at CBM. A light electronic effective mass with *s*-like CBM can increase electrical conductivity. Therefore, the electronic effective mass of the lowest-energy conduction bands plays an important role in determining electrical conductivity of electrode materials. As a result, developing an efficient method to calculate electronic effective mass of these bands is of vital importance to screen doping atoms and evaluate their effectiveness in order to improve electrical conductivity. Based on a concept of solid-state physics, the inverse effective mass tensor can be described as:

(6) $${\text{M}}_{\beta ,u}^{-1}\left(i,k\right)=\frac{1}{\hbar^{2}}\frac{\partial^{2}\varepsilon_{i,k}}{\partial k_{\beta }\partial k_{\mu }}$$

where *ħ* is reduced Planck constant, *k*_*β,μ*_ is reciprocal lattice vector and *ε*_*i,k*_ is the energy of the *i*th band at *k* point. The effective mass of electrons in battery materials can be estimated via parabolic fitting of the actual ɛ-k diagram around the CBM. Although a computational criterion to determine the doping element is quantitatively established to improve electrical conductivity, an accurate calculation for band structure is not easy. Therefore, developing advanced computational methods to realize fast and accurate band structure plays an important role in high-throughput screening to determine doping elements.

In addition, the doping method was used to regulate discharge voltage of cathode materials. In 1998, Goodenough et al. reported that the Fe^2+^/Fe^3+^ and Fe^3+^/Fe^4+^ and V^2+^/V^3+^ redox positions in Fe-SO_4_ and Fe-PO_4_ systems could be tuned by doping [59]. In 2003, Shi et al. [60,61] performed DFT calculations for M-doped LiMn_2_O_4_ (M = Cr, Fe, Co, Ni) cathode material by substituting Mn sites. They revealed that these dopants can generate new O-2p bands resulting in a higher discharge voltage. Therefore, appropriate doped elements in cathode materials can be screened according to calculated discharge voltage. Based on first-principles thermodynamic calculations, the discharge voltage can be calculated, taking LiMO_2_ (M = Co, Mn, Ni) as example, by the following equation:

(7) $$\;\;V^{cell}=-\left(G_{Li_{x+n}MO_{2}}-G_{Li_{xMO_{2}}}-nG_{Li}\right)/n$$

where $G_{Li_{x+n}MO_{2}}$, $G_{Li_{xMO_{2}}}$, *G*_*Li*_ are free energies, *n* is the number of removed Li per unit volume.

The Li-ion migration kinetics also can be tuned by doping strategy. However, inappropriate doping may block the channel of Li-ion migration, leading to poor Li-ion migration performance. The Monte Carlo simulation for Cr-doped LiFePO_4_ indicated that Cr^3+^ ion was not very facile to migrate and block one-dimension channel of Li-ion migration [62,63]. These computations are consistent with corresponding experimental reports [64–67].

Extending a low-concentration doping to a higher-concentration mixture of elements, many solid-solution materials have been synthesized as electrodes and solid electrolytes. These materials may generate an improved electrochemical performance in rate performance and storage capacities. Based on DFT computational modeling, Kang et al. [68] identified that Li[Mn_0.5_Ni_0.5_]O_2_ cathode material had a higher rate capacity than commercialized LiCoO_2_. After that, this type of composited strategy was extensively applied to layered battery materials such as Li[Mn_x_Ni_y_Co_z_]O_2_ (x+y+z ≤ 1) [69,70]. However a series of computational and experimental studies indicated that this composited Li[Mn_0.5_Ni_0.5_]O_2_ was not very stable because Li^+^ and Ni^2+^ exchange each other during the charging process [69,70]. In fact, Ni^2+^ occupations on Li^+^ sites are unfavorable for Li-ion diffusion. In Li[Mn_x_Ni_y_Co_z_]O_2_ (x+y+z<1) materials, Li^+^ occupies transition metal layer. Many experimental and computational studies indicated that a high voltage charge usually leads to oxygen loss structural rearrangement and overcapacity [71–75]. Therefore, improved structural stability of Li[Mn_x_Ni_y_Co_z_]O_2_ (x+y+z<1) materials by avoiding O_2_ evolution plays an important role in their electrochemical applications.

Accurate calculations of Li-ion migration barriers are helpful for selecting suitable doped or mixed elements to improve discharge and charge rates. The transition state method or the molecular dynamics simulations have been extensively applied in calculating energy barriers in materials. However, high-throughput calculations for energy barriers of ionic transports are time consuming. Very recently, Xiao et al. [76] developed a fast computational method by combining bond-valence (BV) and DFT techniques to calculate Li-ion migration barriers. By using this computational method, high-throughput calculations for Li-ion migration barriers of more than 1000 compounds as solid electrolyte were performed to screen superionic conductors in batteries. It is expected that this method also can be used in Li-ion migration barrier calculations in similar electrode materials.

## 6. Interface engineering

The formation of a stable electrolyte-electrode interface (EEI) layer which can make Li^+^ conduction and electronic insulation is critical to ensure high coulombic efficiency, cyclic life, and safety. In the past 10 years, the mosaic model of EEI layers is well accepted to describe interfacial structure, leading to a better understanding of interfacial reactions [77]. The layer results mostly from nucleophilic reactions driven by reactive species of electrode surface to attack electrolyte molecules. The chemical evolution may lead to a gradual growth of EEI layers, accompanied with decomposition of electrode materials.

Very recently, Zhu et al. [78] performed first-principles calculations to evaluate thermodynamic stability of the interfaces between solid electrodes and electrolytes. Their calculations identified that the strong thermodynamic driving force for decomposition at the interfaces limits electrochemical window of solid-electrolyte and the poor chemical compatibility between them. Currently, the interfacial coating layer materials have been extensively applied to enhance electrochemical and chemical stability of interface.

Under an electrochemical environment, the EEI may donate electrons to electrolyte molecules and the stability of interface between the electrode and inflammable electrolyte therefore become correlated moments with safety. As a result, it is necessary to establish the relationship between thermodynamic stability of EEI layer and charge voltages as follows:

$$\Delta G=E_{eq}-E_{interface}\left(x\right)-\Delta N_{Li}\left(\mu_{Li}-eV_{c}\right)$$

where *E*_*eq*_ is the final energy of a certain step of the interfacial equilibrium system, *E*_*interface*_(*x*)  =  *x* ⋅ *E*_*electrolyte*_ + (1 – *x*) ⋅ *E*_*electrode*_ is the initial energy of the interfacial EEI system and *x* is the proportion of electrolyte to the interfacial EEI system, *ΔN*_*Li*_ is the number of the removed Li, *μ*_*Li*_ represents the chemical potentials of bulk Li, *V*_*c*_ is the electromotive force corresponding to charge voltage. As a result, Δ*G* presents the decomposition reaction energy, which defines the stability of the EEI system. However, in terms of high-throughput calculations, it faces a great challenge to develop a quantitative model to describe interfacial stability correlated with the decomposition reaction energy and charge voltage. Composition evolution resulting from interfacial chemical reactions is so complicated that it is difficult to quantitatively describe the stable structure.

**Figure 6.**
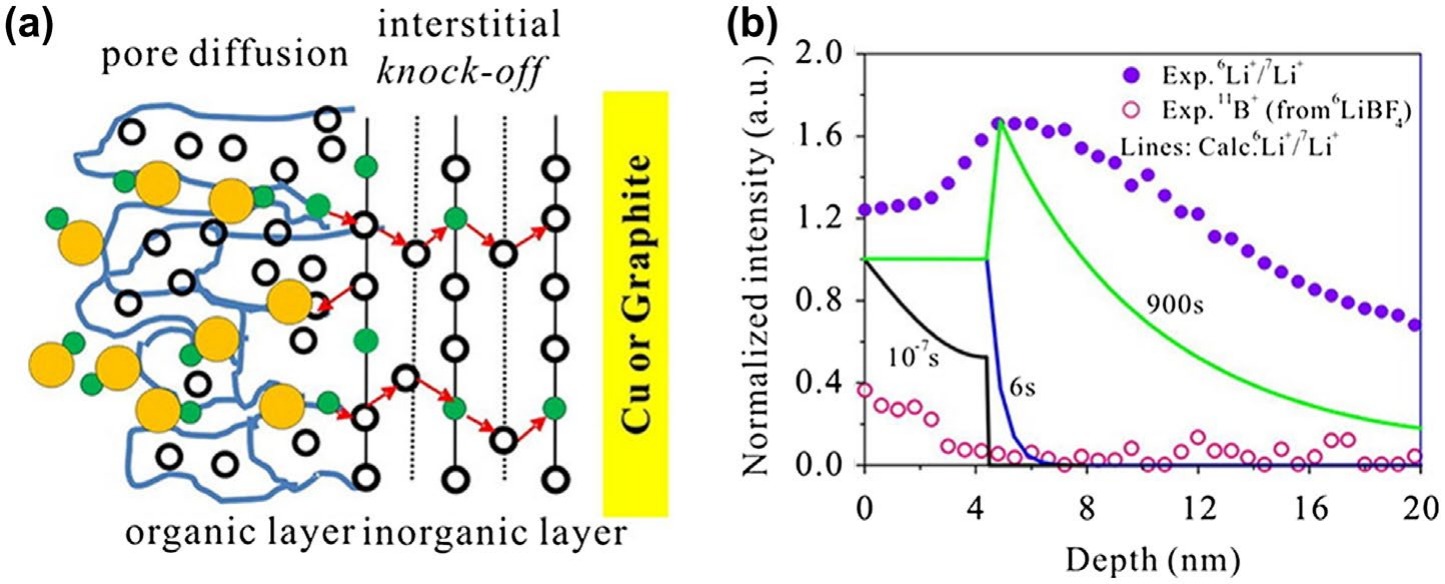
(a) Schematic drawing of pore diffusion in the porous organic layer of EEI and knock-off diffusion in the dense inorganic layer of EEI. The open circles represent the Li^+^ in the EEI. In the porous organic layer, the blue solid lines denote channels through which Li^+^ in the electrolyte (green filled circles) transports with anions (yellow filled circles) via pore diffusion. The red arrows denote that only Li^+^ can diffuse in the dense inorganic layer via the knock-off mechanism. (b) TOF-SIMS measured (by the isotope exchange experiment) depth profiles of ^6^Li^+^/^7^Li^+^ and ^11^B^+^ (symbols) for the EEI growing on a Cu substrate after 900 s soaking and calculated depth profiles of ^6^Li^+^/^7^Li^+^ (solid lines) after 10^−7^, 6, and 900 s soaking. Reproduced from [80] with permission of 2012 American Chemical Society.

The Li-ion migration mechanism in the interface is an important research field with the aim to solve slower kinetics than those in electrolyte and electrode. Smith et al. [79] performed molecular dynamic simulation for LiFePO_4_ (010) in contact with electrolytes and found that the amount of Li^+^ ions could be reduced owing to the accumulation of positive charges at the interfaces. A typical interface structure is the EEI structure formed between electrode and electrolyte. Unravelling the Li^+^ diffusion mechanism in the passivated film plays an important role in optimizing battery performances such as cyclic life and discharge/charge current density. Based on the time-of-flight secondary-ion mass spectrometry (TOF-SIMS) results of Lu and Harris [89], Shi et al. [80] built an EEI model consisting of porous (outer) organic and dense (inner) inorganic layers of Li_2_CO_3_. Using the DFT method, they determined a pore diffusion in outer layer and knock-off diffusion in the inner layer, called the two layer/two mechanism model, as shown in Figure 6. The new model was formulated by using mesoscale diffusion equations and predicted the unusual isotope ratio ^6^Li^+^/^7^Li^+^ profile measured by TOF-SIMS, which increases from the EEI/electrolyte surface and peaks at a depth of 5 nm, and then gradually decreases within the dense layer.

## 7. Design of novel battery materials based on structure–properties relationships

In recent years, high-throughput calculations to screen novel functional materials attracted extensive attention due to the influence of integrated computational material engineering (ICME) and the MGI [81–85]. In 2011, Ceder et al. combined the Inorganic Crystal Structure Database (ICSD) with high-throughput calculations to screen thousands of Li-ion battery materials by setting the values of energy capacity, voltage, and volume change [81, 86, 87]. However, it was shown that these screened cathode materials cannot meet the requirement of practical applications. The screening must consider the suitable physical and chemical parameters, such as disordered structure, nanostructure, size, doping, and surface and interfacial structures, to describe structural stability.

**Figure 7.**
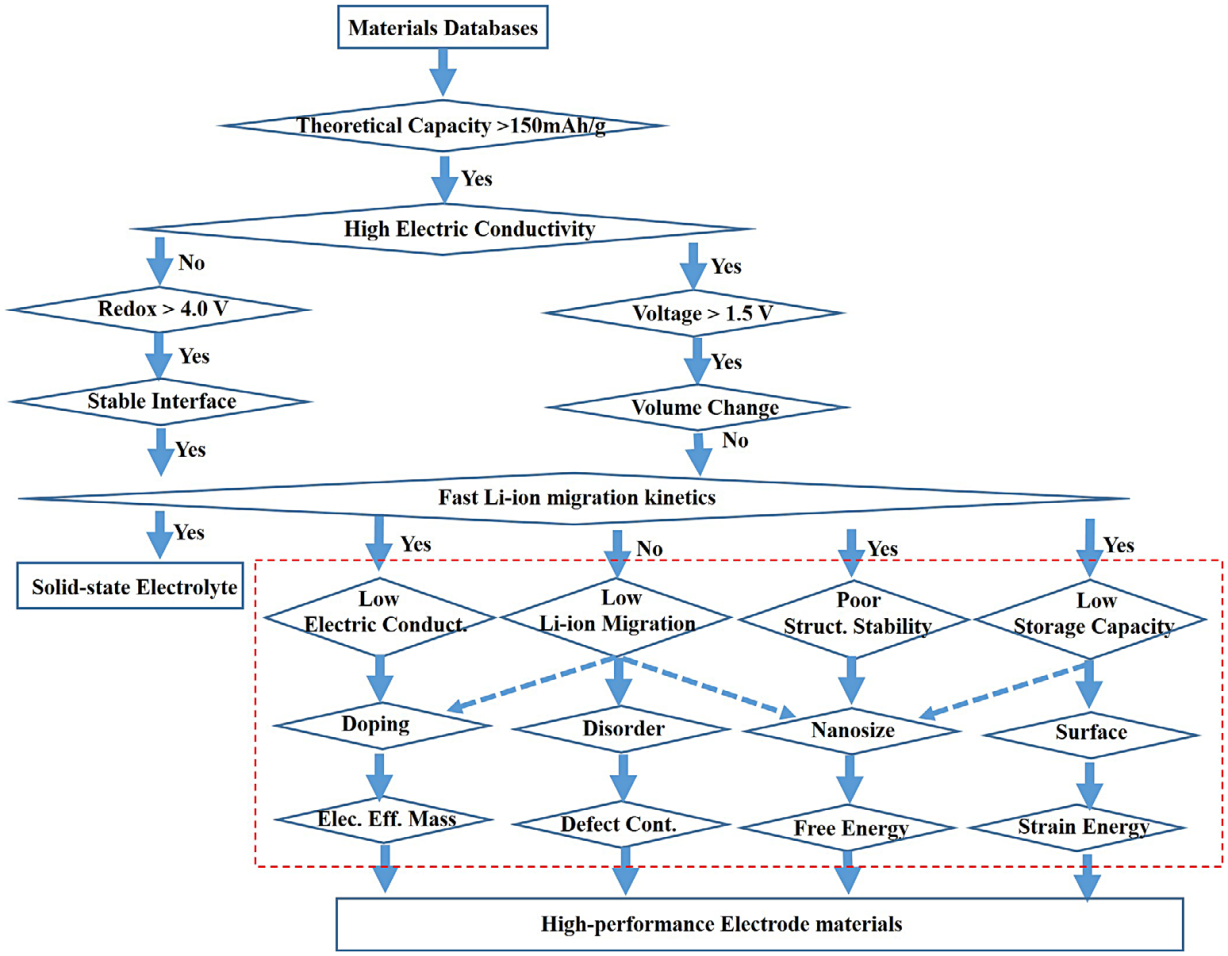
Flow chart of high-throughput calculations with different computational level in accuracy to screen high-performance electrode and solid electrolyte materials. The dashed rectangle is introduced in this review. The theoretical capacity, voltage, and redox are set to 150 mA h g^–1^, 1.5 V, and 4.0 V, respectively, as an example.

It is important to develop an efficient strategy to perform high-throughput materials screening calculations. Based on the above analysis, we established a flow chart of high-throughput calculations with different accuracies to screen high-performance electrode and solid electrolyte materials (Figure 7). Because some structure–property relationships such as interface-correlated Li-ion stability and Li-ion migration have not been quantitatively established, they are not included in the flow chart. High-throughput calculations highlighted by the dashed line in Figure 7 to optimize electrochemical performances of battery materials are presented based on structure–property relationships which are summarized in the present review.

As shown in Figure 7, electrical conductivity and Li-ion migration kinetics are two important parameters for evaluating the electrochemical performance of solid electrolyte and electrode materials. A solid electrolyte should have an extremely low electrical conductivity, but a high Li-ion migration rate. In contrast, an electrode material should exhibit both a high electrical conductivity and fast Li-ion kinetics. We can make further second-order corrections to the electrochemical performance of electrode materials based on the discussions in this review, as shown in the red rectangle in Figure 7.

Direct high-throughput calculations for some physical and chemical properties such as Li-ion migration barriers and size-dependent structural stability are expensive. Therefore, a fast computational method with acceptable accuracy is necessary to perform high-throughput calculations for battery materials. Because the structure–property relationships do not have a one-to-one correlation, one must determine the most likely strategy to obtain the most efficient performance optimization.

By interfacing with the ICSD database, Xiao et al. [88] developed a semi-empirical BV method to perform high-throughput calculations to screen solid electrolyte materials with low Li-ion migration barriers. The electrical conductivity related to band structure was calculated by the first-principles method. Therefore, it is necessary to develop computational methods with different accuracies to carry out high-throughput calculations, for example, Li-ion migration kinetics calculated by the semi-empirical method, and band structure calculated by the first-principles method [76].

## 8. Concluding remarks and outlook

In this review, we have illustrated the quantitative description of structure–property relationships. These parameters in high-throughput calculations play an important role in discovering new materials and optimizing material performances. For example, defect concentration can be applied as a screening criterion to determine the effect of the defect on accelerating the Li-ion migration kinetics. Electron effective masses may be a screening criterion for choosing doped elements to improve electrical conductivity. One must determine the most likely structural characters to obtain the most efficient performance optimization, as many structure–property relationships do not have a one-to-one correlation.

In terms of computational and experimental methods, developing more efficient multiscale computational models with different computational accuracies will be essential for an appropriate description of battery materials. In addition, developing synchrotron and *in situ* experimental characterization techniques for structures and electrochemical properties is significantly important to understand dynamic mechanisms of discharge and charge processes.

## Disclosure statement

No potential conflict of interest was reported by the authors.
